# Supplementary material for: Adjuvants MPLA and SMNP induce antiviral immunity and indirectly revert HIV-1 latency
Source: PLoS One. 2026 Jul 20;21(7):e0348959. doi: 10.1371/journal.pone.0348959 (PMC13384302; doi:10.1371/journal.pone.0348959)
Supplement: S1 Table — (PDF) [file pone.0348959.s001.pdf]

S1 Table. EC50 values for both SMNP and MPLA of costimulatory markers and cytokines.

μg/mL	CD80	CD83	CD86	IL-6	IL-10	IL-12	TNFα
MPLA	2.27	12.67	2.80	2.15	-	2.13	7.27
SMNP	0.65	1.82	0.65	1.00	20.65	9.83	0.62
