# Supplementary material for: Adjuvants MPLA and SMNP induce antiviral immunity and indirectly revert HIV-1 latency
Source: PLoS One. 2026 Jul 20;21(7):e0348959. doi: 10.1371/journal.pone.0348959 (PMC13384302; doi:10.1371/journal.pone.0348959)
Supplement: S1 Fig — (PDF) [file pone.0348959.s002.pdf]

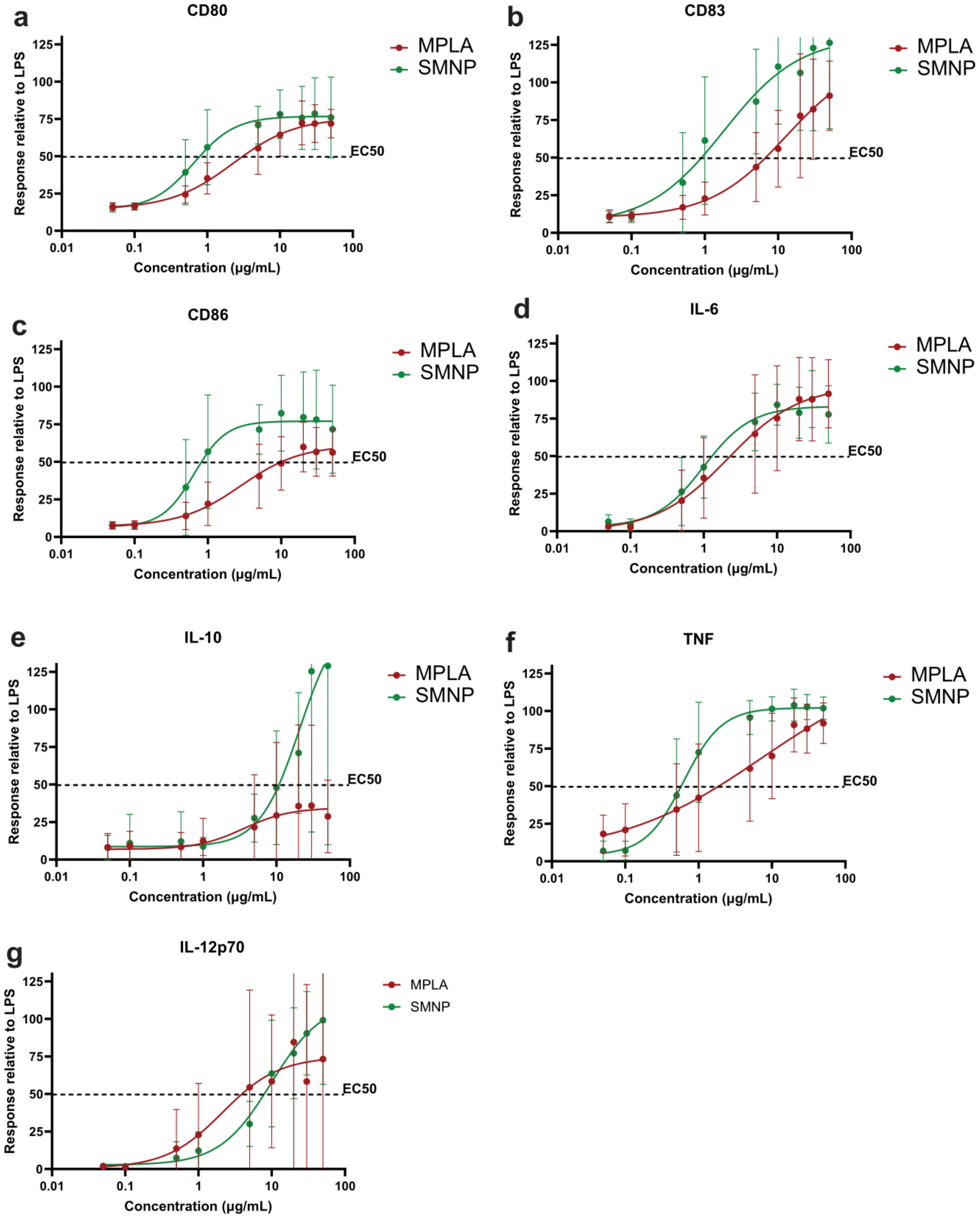

S1 Figure. EC50 value calculations for SMNP and MPLA for costimulatory markers and cytokines. DCs from six donors were stimulated with increasing doses of SMNP or MPLA (0·05 – 0·1 – 0·5 – 1 – 5 – 10 – 20 – 30 – 50µg/mL) and responses were normalized to the LPS positive control. (A-G) Logistic regression plots are displayed for obtained normalised values, EC50 values were calculated using a four-parameter logistic (4PL) model and displayed in table 2.
