# Supplementary material for: Adjuvants MPLA and SMNP induce antiviral immunity and indirectly revert HIV-1 latency
Source: PLoS One. 2026 Jul 20;21(7):e0348959. doi: 10.1371/journal.pone.0348959 (PMC13384302; doi:10.1371/journal.pone.0348959)
Supplement: S2 Fig — (PDF) [file pone.0348959.s003.pdf]

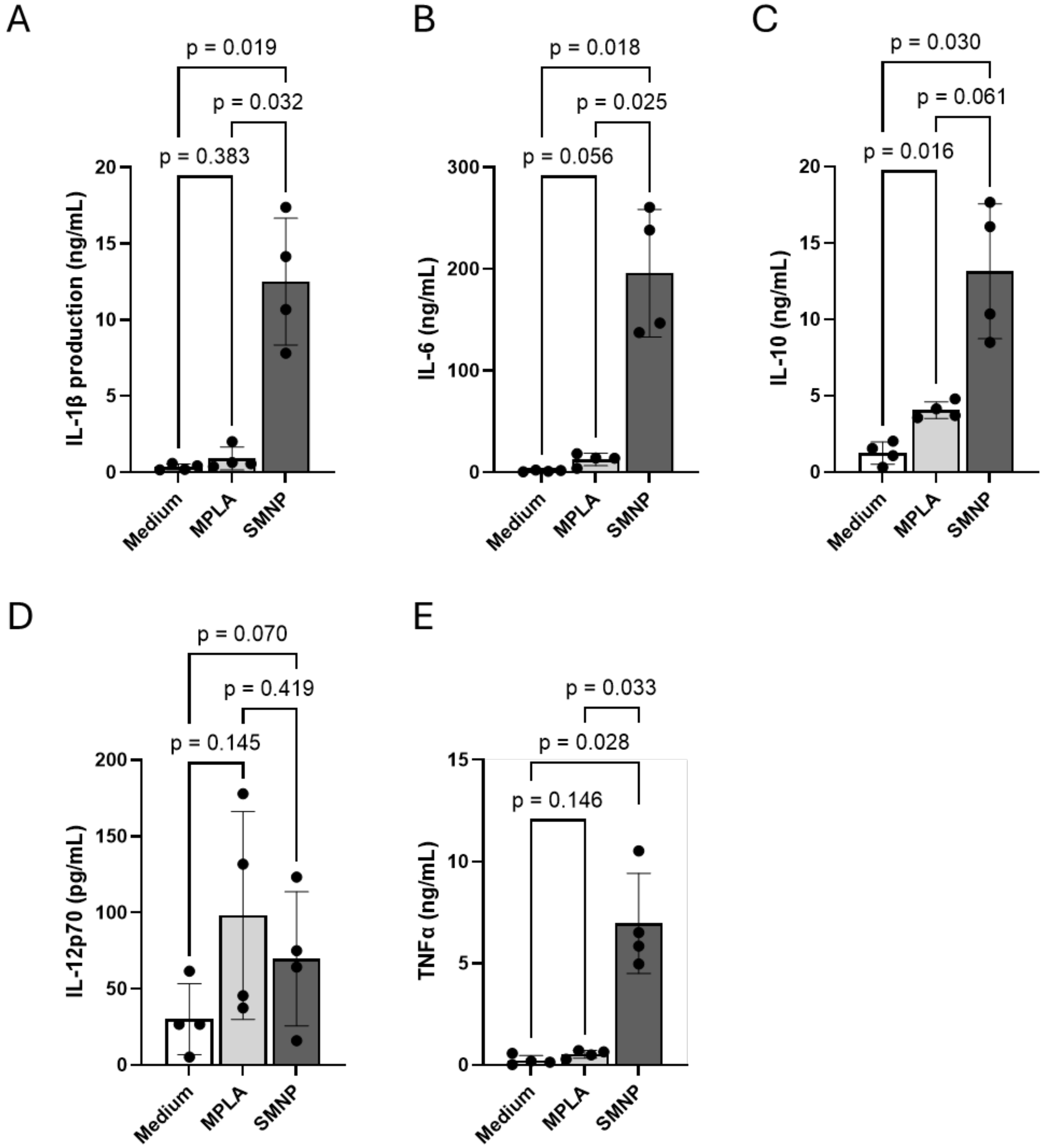

S2 Figure. SMNP and MPLA cytokine induction in PBMC of healthy donors. SMNP induced potent cytokine responses in PBMCs, whereas MPLA did not. Comparisons between MPLA, SMNP and medium control were performed using a mixed-effects analysis. P-values were adjusted for multiple comparison using Tukey method.
