# Supplementary material for: Adjuvants MPLA and SMNP induce antiviral immunity and indirectly revert HIV-1 latency
Source: PLoS One. 2026 Jul 20;21(7):e0348959. doi: 10.1371/journal.pone.0348959 (PMC13384302; doi:10.1371/journal.pone.0348959)
Supplement: S3 Fig — (PDF) [file pone.0348959.s004.pdf]

A.

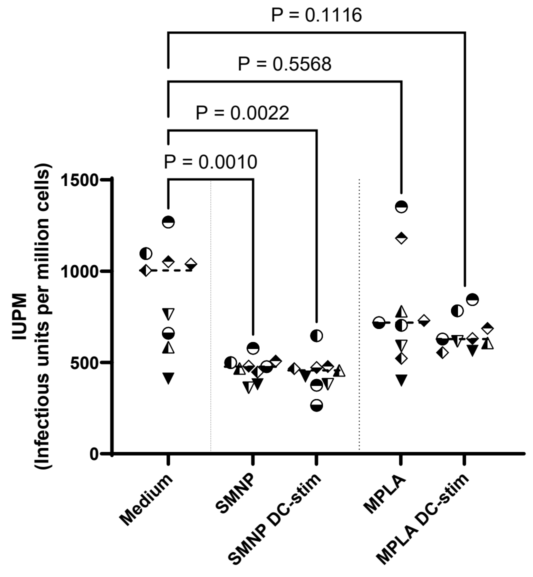

B.

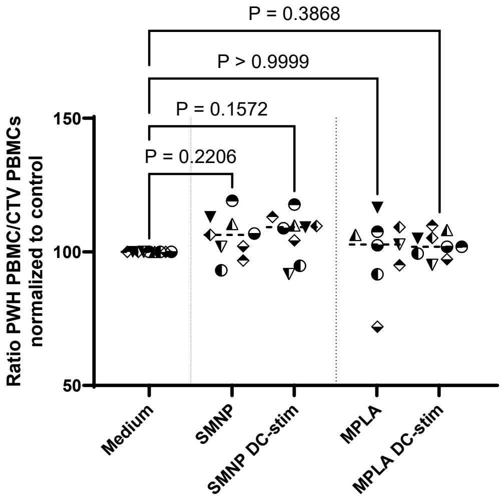

S3 Figure. Effect of SMNP and MPLA induced cytokine production on the HIV reservoir and cell survival. Latency reversal is facilitated by a pro-inflammatory cytokine milieu. (A) IUPM after treatment with the compounds for each participant (n=9); the median IUPM is displayed. Comparisons of each condition to medium control were made using a one way ANOVA. Conditions are: unstimulated medium control, SMNP stimulation of PBMCs, supernatant from DCs stimulated for 24h with SMNP, MPLA stimulation of PBMCs, supernatant from DCs stimulated for 24h with MPLA. (B) Ratio of PWH PBMCs/healthy Cell trace violet (CTV)-stained PBMCs normalized to the medium control to check cytotoxicity of SMNP and MPLA. Comparisons of each condition to control were made using a Friedman test, * P < 0·05, **P < 0·01.
