# Supplementary material for: Adjuvants MPLA and SMNP induce antiviral immunity and indirectly revert HIV-1 latency
Source: PLoS One. 2026 Jul 20;21(7):e0348959. doi: 10.1371/journal.pone.0348959 (PMC13384302; doi:10.1371/journal.pone.0348959)
Supplement: Attachment — Submitted filename: Response to Reviewers.docx [file pone.0348959.s007.docx]

**Journal Requirements and response to editoral comments:**

1. Please ensure that your manuscript meets PLOS ONE's style requirements, including those for file naming.

**Response:** Style requirements have been incorporated.

2. Thank you for stating the following financial disclosure:

“This research was funded by Health~Holland/Aidsfonds(LSHM19101/P-44802) (TG/NK), Health~Holland/Amsterdam UMC(2019-1167) (TG/NK), ZonMW/Aidsfonds(446002508) (TG/GdB), Aids-fonds/NWO (KICH2.V4P.AF23.001) (TG/NK/GdB/RS).”

Please state what role the funders took in the study.  If the funders had no role, please state: "The funders had no role in study design, data collection and analysis, decision to publish, or preparation of the manuscript." If this statement is not correct you must amend it as needed.

Please include this amended Role of Funder statement in your cover letter; we will change the online submission form on your behalf.

**Response:** We adjusted the financial disclosure as one of our grants was missing. We confirm that the funders had no role in the study as mentioned in the statement:

This research was funded by Health~Holland/Aidsfonds(LSHM19101/P-44802) (TBHG/NAK), Health~Holland/Amsterdam UMC(2019-1167) (TBHG/NAK), ZonMW/Aidsfonds(446002508) (TBHG/GJdB), Aids-fonds/NWO (KICH2.V4P.AF23.001) (TBHG/NAK/GJdB/RWS), Aidsfonds (P-66401) (GJdB/NAK/TBHG). The funders had no role in study design, data collection and analysis, decision to publish, or preparation of the manuscript.

3. We note that your Data Availability Statement is currently as follows: “All data are reported in a submitted manuscript.” Please confirm at this time whether or not your submission contains all raw data required to replicate the results of your study.

**Response:** We have added the raw data in a supplementary file (S1 data file). This file contains all measurements behind the graphs displayed in the manuscript figures and supplementary figures.

4. If the reviewer comments include a recommendation to cite specific previously published works, please review and evaluate these publications to determine whether they are relevant and should be cited. There is no requirement to cite these works unless the editor has indicated otherwise. 
**Response:** Not Applicable.

5 Please review your reference list to ensure that it is complete and correct. Any changes to the reference list should be mentioned in the rebuttal letter that accompanies your revised manuscript.

**Response:** No changes applicable. However, there are a few changes in the reference order due revisions in the manuscript.

6. Please include captions for your Supporting Information files at the end of your manuscript, and update any in-text citations to match accordingly.

**Response:** Captions for supporting information has been added.

**Additional Editor Comments:**
The authors need to address the reviewers specific comments especially around statistical analysis and data interpretation

**Response:** We have adjusted the manuscript and statistical analysis as suggested by the reviewer.

**Response to reviewers' comments:
Response:** We thank the reviewers for the thorough evaluation of our manuscript and the very helpful comments to improve our manuscript. Below is a point-by-point response to the reviewers’ comments and description of changes made to the manuscript.

**Reviewer #1:** This manuscripts evaluates the effects of the vaccine adjuvants MPLA and SMNP on immune activation and inducible HIV reservoir in people with HIV on suppressive ART. SMNP is a new adjuvant that has been evaluated in vaccine studies in healthy volunteers and will also be part of therapeutic vaccine studies. The authors performed as series of in vitro studies and showed that both adjuvants activated dendritic cells through TLR4 signaling and induced pro-inflammatory cytokine production. SMNP was superior to MPLA. Interestingly, SMNP, but not MPLA, reduced the size of the inducible HIV reservoir ex vivo without detectable bystander cytotoxicity, likely through cytokine-mediated latency reversal and immune activation. These findings support further investigation of SMNP as a potential adjuvant for therapeutic HIV vaccination strategies. The data is presented clearly and it relevant to the vaccine field and HIV in particular as several ongoing and planned studies will use SMNP as the adjuvant.

Studies in healthy volunteers and pre-clinical studies shows that SMNP is a strong adjuvant that improves immunogenicity of different protein vaccines. The data of PWH is also reassuring that despite immune dysregulation activation of dendritic cells occur. HIV-specific immune responses were not directly evaluated in this study. The ex vivo data presented show that SMNP-mediated immune activation and cytokine-mediated latency reversal are not associated with cytotoxicity. However it’s impact on HIV-specific immune responses was not evaluated. It would strengthen the paper to include such evaluations, if possible.

**Response:** We agree with the reviewer, however due to technical limitations and limited sample availability this analysis has not been performed. Vaccine adjuvants are used to trigger innate response to boost recognition and triggering of downstream adaptive response against an antigen. Here we show that the adjuvants are capable of triggering an innate response using PBMC from PWH. This immune activation may also boost the autologous HIV-specific T cell response, however this is hard to determine in vitro, as peptide pools stimulation is the gold standard for the analysis of HIV-specific T cell responses bypassing major components of the innate immune response. The synthetic peptides pools are already processed thus skipping the complex intracellular processing pathways and innate danger-sensing mechanisms (like TLR and inflammasome activation) required for native proteins in vaccines.

Since effects on the HIV reservoir would be of interest in the context of therapeutic vaccination, the following statement in the abstract “However, as reservoir reduction was observed ex vivo, further evaluation is warranted to ensure safe use of such adjuvants in PWH” in unclear. It has been shown in studies of vaccination or co-infection that while viral blips may occur, during ART viral suppression is not compromised. The authors should clarify the statement about safety in the context of HIV and the broader goals of remission strategies.

**Response:** We have removed this sentence from the abstract, as this is not really addressed in this manuscript. We have included this in the introduction (line 90-93) and discussion (line 358-365).

**Reviewer #2:** The manuscript addresses whether MPLA and SMNP adjuvants induce innate immune activation in ART-suppressed PWH and if this lowers the inducible HIV-1 reservoir ex vivo. The conclusion that SMNP is more immunostimulatory than MPLA is supported by the activation and cytokine data and the observation that SMNP reduces inducible reservoir ex vivo has potential. The authors appropriately acknowledge that these are ex vivo findings requiring further evaluation before clinical translation. I ask that they also consider the following comments.

Major Comments

1. The statistical analysis section is too vague and not the best approach for the experimental design. The authors state that two-way ANOVA, one-way ANOVA, Brown-Forsythe/Welch ANOVA, and post-hoc corrections were used, but they do not clearly specify which test was used for each figure, whether donor-matched repeated-measures analyses were used, what correction was applied for each. Since many comparisons involve repeated measures across doses or treatments within the same donors, paired repeated-measures models or mixed-effects models would be more appropriate than simple ANOVA. Exact p values should be reported where possible and figure legends should clearly state the statistical test, n, and correction method for each panel.
**Response:** We have adjusted the statistical analysis as suggested by the reviewer and used a mixed-effect model to compare the different conditions within groups. This information has been added to the M&M section (line 187-191) and the legends of the figure. The exact p-values have been added to the figures and the number of donors/PWH tested is given in the figure legends.

2. I recommend more conservative language around reservoir reduction. The data suggest reduced inducible virus after SMNP exposure, but the mechanism is inferred rather than directly shown. The authors should avoid implying selective elimination of infected cells unless supported by additional evidence. Alternative explanations, including altered inducibility, assay interference, cytokine-mediated effects on outgrowth/readout, or changes in target-cell activation should be more explicitly discussed.

**Response:** We have adjusted our wording with regards to reservoir reduction throughout the manuscript and added alternative explanations for the observed reduction to the discussion (line 339-342).

3. The manuscript language is clear but would benefit from copy editing. There are multiple grammatical errors, spacing/formatting issues, and imprecise phrasing, including “ex vivowithout,” “supressed,” “monoplo,” and several awkward sentences in the Introduction and Discussion. These do not prevent interpretation but should be corrected.

**Response:** We have copy-edited the manuscript and corrected mistakes throughout the manuscript. We have rephrased parts in the introduction, results and discussion (line 57-64, 67-69, 213-216, 228-231, 240-244, 258-259, 269-275, 277, 281-283, 296-300, 314-319).
