# Supplementary material for: Adjuvants MPLA and SMNP induce antiviral immunity and indirectly revert HIV-1 latency
Source: PLoS One. 2026 Jul 20;21(7):e0348959. doi: 10.1371/journal.pone.0348959 (PMC13384302; doi:10.1371/journal.pone.0348959)
Supplement: Peer review history

# Peer review: Decision Letter 0

**DOI:** 10.1371/journal.pone.0348959.r001

**Authors:** Alan Landay

5 Jun 2026

PONE-D-26-18832

Adjuvants MPLA and SMNP induce antiviral immunity and indirectly revert HIV-1 latency

PLOS One

Dear Dr. Koostra,

Thank you for submitting your manuscript to PLOS ONE. After careful consideration, we feel that it has merit but does not fully meet PLOS ONE’s publication criteria as it currently stands. Therefore, we invite you to submit a revised version of the manuscript that addresses the points raised during the review process.

Please submit your revised manuscript by Jul 19 2026 11:59PM. If you will need more time than this to complete your revisions, please reply to this message or contact the journal office at plosone@plos.org. When you're ready to submit your revision, log on to https://www.editorialmanager.com/pone/ and select the 'Submissions Needing Revision' folder to locate your manuscript file.

Please include the following items when submitting your revised manuscript:

- A letter that responds to each point raised by the academic editor and reviewer(s). You should upload this letter as a separate file labeled 'Response to Reviewers'.
- A marked-up copy of your manuscript that highlights changes made to the original version. You should upload this as a separate file labeled 'Revised Manuscript with Track Changes'.
- An unmarked version of your revised paper without tracked changes. You should upload this as a separate file labeled 'Manuscript'.

If you would like to make changes to your financial disclosure, please include your updated statement in your cover letter. Guidelines for resubmitting your figure files are available below the reviewer comments at the end of this letter.

If applicable, we recommend that you deposit your laboratory protocols in protocols.io to enhance the reproducibility of your results. Protocols.io assigns your protocol its own identifier (DOI) so that it can be cited independently in the future. For instructions see: https://journals.plos.org/plosone/s/submission-guidelines#loc-laboratory-protocols. Additionally, PLOS ONE offers an option for publishing peer-reviewed Lab Protocol articles, which describe protocols hosted on protocols.io. Read more information on sharing protocols at https://plos.org/protocols?utm_medium=editorial-email&utm_source=authorletters&utm_campaign=protocols.

As the corresponding author, your ORCID iD is verified in the submission system and will appear in the published article. PLOS supports the use of ORCID, and we encourage all coauthors to register for an ORCID iD and use it as well. Please encourage your coauthors to verify their ORCID iD within the submission system before final acceptance, as unverified ORCID iDs will not appear in the published article. Only the individual author can complete the verification step; PLOS staff cannot verify ORCID iDs on behalf of authors.

We look forward to receiving your revised manuscript.

Kind regards,

Alan Landay

Academic Editor

PLOS One

Journal Requirements:

When submitting your revision, we need you to address these additional requirements.

1. Please ensure that your manuscript meets PLOS ONE's style requirements, including those for file naming. The PLOS ONE style templates can be found at

https://journals.plos.org/plosone/s/file?id=wjVg/PLOSOne_formatting_sample_main_body.pdf and

https://journals.plos.org/plosone/s/file?id=ba62/PLOSOne_formatting_sample_title_authors_affiliations.pdf

2. Thank you for stating the following financial disclosure:

“This research was funded by Health~Holland/Aidsfonds(LSHM19101/P-44802) (TG/NK), Health~Holland/Amsterdam UMC(2019-1167) (TG/NK), ZonMW/Aidsfonds(446002508) (TG/GdB), Aids-fonds/NWO (KICH2.V4P.AF23.001) (TG/NK/GdB/RS).”

Please state what role the funders took in the study.  If the funders had no role, please state: "The funders had no role in study design, data collection and analysis, decision to publish, or preparation of the manuscript."

If this statement is not correct you must amend it as needed.

Please include this amended Role of Funder statement in your cover letter; we will change the online submission form on your behalf.

3. We note that your Data Availability Statement is currently as follows: “All data are reported in a submitted manuscript.”

Please confirm at this time whether or not your submission contains all raw data required to replicate the results of your study. Authors must share the “minimal data set” for their submission. PLOS defines the minimal data set to consist of the data required to replicate all study findings reported in the article, as well as related metadata and methods (https://journals.plos.org/plosone/s/data-availability#loc-minimal-data-set-definition).

For example, authors should submit the following data:

- The values behind the means, standard deviations and other measures reported;

- The values used to build graphs;

- The points extracted from images for analysis.

Authors do not need to submit their entire data set if only a portion of the data was used in the reported study.

If your submission does not contain these data, please either upload them as Supporting Information files or deposit them to a stable, public repository and provide us with the relevant URLs, DOIs, or accession numbers. For a list of recommended repositories, please see https://journals.plos.org/plosone/s/recommended-repositories.

If there are ethical or legal restrictions on sharing a de-identified data set, please explain them in detail (e.g., data contain potentially sensitive information, data are owned by a third-party organization, etc.) and who has imposed them (e.g., an ethics committee). Please also provide contact information for a data access committee, ethics committee, or other institutional body to which data requests may be sent. If data are owned by a third party, please indicate how others may request data access.

4. If the reviewer comments include a recommendation to cite specific previously published works, please review and evaluate these publications to determine whether they are relevant and should be cited. There is no requirement to cite these works unless the editor has indicated otherwise.

5 Please review your reference list to ensure that it is complete and correct. If you have cited papers that have been retracted, please include the rationale for doing so in the manuscript text, or remove these references and replace them with relevant current references. Any changes to the reference list should be mentioned in the rebuttal letter that accompanies your revised manuscript. If you need to cite a retracted article, indicate the article’s retracted status in the References list and also include a citation and full reference for the retraction notice.

6. Please include captions for your Supporting Information files at the end of your manuscript, and update any in-text citations to match accordingly. Please see our Supporting Information guidelines for more information: http://journals.plos.org/plosone/s/supporting-information.

Additional Editor Comments :

The authors need to address the reviewers specific comments especially around statistical analysis and data interpretation

[Note: HTML markup is below. Please do not edit.]

Reviewers' comments:

Reviewer's Responses to Questions

**Comments to the Author**

1. Is the manuscript technically sound, and do the data support the conclusions?

The manuscript must describe a technically sound piece of scientific research with data that supports the conclusions. Experiments must have been conducted rigorously, with appropriate controls, replication, and sample sizes. The conclusions must be drawn appropriately based on the data presented.

Reviewer #1: Yes

Reviewer #2: Yes

**********

2. Has the statistical analysis been performed appropriately and rigorously?

Reviewer #1: I Don't Know

Reviewer #2: No

**********

3. Have the authors made all data underlying the findings in their manuscript fully available?

The PLOS Data policy requires authors to make all data underlying the findings described in their manuscript fully available without restriction, with rare exception (please refer to the Data Availability Statement in the manuscript PDF file). The data should be provided as part of the manuscript or its supporting information, or deposited to a public repository. For example, in addition to summary statistics, the data points behind means, medians and variance measures should be available. If there are restrictions on publicly sharing data—e.g. participant privacy or use of data from a third party—those must be specified.

Reviewer #1: Yes

Reviewer #2: No

**********

4. Is the manuscript presented in an intelligible fashion and written in standard English?

PLOS ONE does not copyedit accepted manuscripts, so the language in submitted articles must be clear, correct, and unambiguous. Any typographical or grammatical errors should be corrected at revision, so please note any specific errors here.

Reviewer #1: Yes

Reviewer #2: Yes

**********

5. Review Comments to the Author

Please use the space provided to explain your answers to the questions above. You may also include additional comments for the author, including concerns about dual publication, research ethics, or publication ethics. (Please upload your review as an attachment if it exceeds 20,000 characters)

Reviewer #1: This manuscripts evaluates the effects of the vaccine adjuvants MPLA and SMNP on immune activation and inducible HIV reservoir in people with HIV on suppressive ART. SMNP is a new adjuvant that has been evaluated in vaccine studies in healthy volunteers and will also be part of therapeutic vaccine studies. The authors performed as series of in vitro studies and showed that both adjuvants activated dendritic cells through TLR4 signaling and induced pro-inflammatory cytokine production. SMNP was superior to MPLA. Interestingly, SMNP, but not MPLA, reduced the size of the inducible HIV reservoir ex vivo without detectable bystander cytotoxicity, likely through cytokine-mediated latency reversal and immune activation. These findings support further investigation of SMNP as a potential adjuvant for therapeutic HIV vaccination strategies. The data is presented clearly and it relevant to the vaccine field and HIV in particular as several ongoing and planned studies will use SMNP as the adjuvant.

Studies in healthy volunteers and pre-clinical studies shows that SMNP is a strong adjuvant that improves immunogenicity of different protein vaccines. The data of PWH is also reassuring that despite immune dysregulation activation of dendritic cells occur. HIV-specific immune responses were not directly evaluated in this study. The ex vivo data presented show that SMNP-mediated immune activation and cytokine-mediated latency reversal are not associated with cytotoxicity. However it’s impact on HIV-specific immune responses was not evaluated. It would strengthen the paper to include such evaluations, if possible.

Since effects on the HIV reservoir would be of interest in the context of therapeutic vaccination, the following statement in the abstract “However, as reservoir reduction was observed ex vivo, further evaluation is warranted to ensure safe use of such adjuvants in PWH” in unclear. It has been shown in studies of vaccination or co-infection that while viral blips may occur, during ART viral suppression is not compromised. The authors should clarify the statement about safety in the context of HIV and the broader goals of remission strategies.

Reviewer #2: The manuscript addresses whether MPLA and SMNP adjuvants induce innate immune activation in ART-suppressed PWH and if this lowers the inducible HIV-1 reservoir ex vivo. The conclusion that SMNP is more immunostimulatory than MPLA is supported by the activation and cytokine data and the observation that SMNP reduces inducible reservoir ex vivo has potential. The authors appropriately acknowledge that these are ex vivo findings requiring further evaluation before clinical translation. I ask that they also consider the following comments.

Major Comments

1. The statistical analysis section is too vague and not the best approach for the experimental design. The authors state that two-way ANOVA, one-way ANOVA, Brown-Forsythe/Welch ANOVA, and post-hoc corrections were used, but they do not clearly specify which test was used for each figure, whether donor-matched repeated-measures analyses were used, what correction was applied for each. Since many comparisons involve repeated measures across doses or treatments within the same donors, paired repeated-measures models or mixed-effects models would be more appropriate than simple ANOVA. Exact p values should be reported where possible and figure legends should clearly state the statistical test, n, and correction method for each panel.

2. I recommend more conservative language around reservoir reduction. The data suggest reduced inducible virus after SMNP exposure, but the mechanism is inferred rather than directly shown. The authors should avoid implying selective elimination of infected cells unless supported by additional evidence. Alternative explanations, including altered inducibility, assay interference, cytokine-mediated effects on outgrowth/readout, or changes in target-cell activation should be more explicitly discussed.

3. The manuscript language is clear but would benefit from copy editing. There are multiple grammatical errors, spacing/formatting issues, and imprecise phrasing, including “ex vivowithout,” “supressed,” “monoplo,” and several awkward sentences in the Introduction and Discussion. These do not prevent interpretation but should be corrected.

**********

6. PLOS authors have the option to publish the peer review history of their article (what does this mean?). If published, this will include your full peer review and any attached files.

If you choose “no”, your identity will remain anonymous but your review may still be made public.

**Do you want your identity to be public for this peer review?**  For information about this choice, including consent withdrawal, please see our Privacy Policy.

Reviewer #1: **Yes:** Marina Caskey

Reviewer #2: No

**********

[NOTE: If reviewer comments were submitted as an attachment file, they will be attached to this email and accessible via the submission site. Please log into your account, locate the manuscript record, and check for the action link "View Attachments". If this link does not appear, there are no attachment files.]

To ensure your figures meet our technical requirements, please review our figure guidelines: https://journals.plos.org/plosone/s/figures

You may also use PLOS’s free figure tool, NAAS, to help you prepare publication quality figures: https://journals.plos.org/plosone/s/figures#loc-tools-for-figure-preparation.

NAAS will assess whether your figures meet our technical requirements by comparing each figure against our figure specifications.

# Author comment: Author response to Decision Letter 1

**DOI:** 10.1371/journal.pone.0348959.r002

11 Jun 2026

Journal Requirements and response to editoral comments:

1. Please ensure that your manuscript meets PLOS ONE's style requirements, including those for file naming.

Response: Style requirements have been incorporated.

2. Thank you for stating the following financial disclosure:

“This research was funded by Health~Holland/Aidsfonds(LSHM19101/P-44802) (TG/NK), Health~Holland/Amsterdam UMC(2019-1167) (TG/NK), ZonMW/Aidsfonds(446002508) (TG/GdB), Aids-fonds/NWO (KICH2.V4P.AF23.001) (TG/NK/GdB/RS).”

Please state what role the funders took in the study. If the funders had no role, please state: "The funders had no role in study design, data collection and analysis, decision to publish, or preparation of the manuscript." If this statement is not correct you must amend it as needed.

Please include this amended Role of Funder statement in your cover letter; we will change the online submission form on your behalf.

Response: We adjusted the financial disclosure as one of our grants was missing. We confirm that the funders had no role in the study as mentioned in the statement:

This research was funded by Health~Holland/Aidsfonds(LSHM19101/P-44802) (TBHG/NAK), Health~Holland/Amsterdam UMC(2019-1167) (TBHG/NAK), ZonMW/Aidsfonds(446002508) (TBHG/GJdB), Aids-fonds/NWO (KICH2.V4P.AF23.001) (TBHG/NAK/GJdB/RWS), Aidsfonds (P-66401) (GJdB/NAK/TBHG). The funders had no role in study design, data collection and analysis, decision to publish, or preparation of the manuscript.

3. We note that your Data Availability Statement is currently as follows: “All data are reported in a submitted manuscript.” Please confirm at this time whether or not your submission contains all raw data required to replicate the results of your study.

Response: We have added the raw data in a supplementary file (S1 data file). This file contains all measurements behind the graphs displayed in the manuscript figures and supplementary figures.

4. If the reviewer comments include a recommendation to cite specific previously published works, please review and evaluate these publications to determine whether they are relevant and should be cited. There is no requirement to cite these works unless the editor has indicated otherwise.

Response: Not Applicable.

5 Please review your reference list to ensure that it is complete and correct. Any changes to the reference list should be mentioned in the rebuttal letter that accompanies your revised manuscript.

Response: No changes applicable. However, there are a few changes in the reference order due revisions in the manuscript.

6. Please include captions for your Supporting Information files at the end of your manuscript, and update any in-text citations to match accordingly.

Response: Captions for supporting information has been added.

Additional Editor Comments:

The authors need to address the reviewers specific comments especially around statistical analysis and data interpretation

Response: We have adjusted the manuscript and statistical analysis as suggested by the reviewer.

Response to reviewers' comments:

Response: We thank the reviewers for the thorough evaluation of our manuscript and the very helpful comments to improve our manuscript. Below is a point-by-point response to the reviewers’ comments and description of changes made to the manuscript.

Reviewer #1: This manuscripts evaluates the effects of the vaccine adjuvants MPLA and SMNP on immune activation and inducible HIV reservoir in people with HIV on suppressive ART. SMNP is a new adjuvant that has been evaluated in vaccine studies in healthy volunteers and will also be part of therapeutic vaccine studies. The authors performed as series of in vitro studies and showed that both adjuvants activated dendritic cells through TLR4 signaling and induced pro-inflammatory cytokine production. SMNP was superior to MPLA. Interestingly, SMNP, but not MPLA, reduced the size of the inducible HIV reservoir ex vivo without detectable bystander cytotoxicity, likely through cytokine-mediated latency reversal and immune activation. These findings support further investigation of SMNP as a potential adjuvant for therapeutic HIV vaccination strategies. The data is presented clearly and it relevant to the vaccine field and HIV in particular as several ongoing and planned studies will use SMNP as the adjuvant.

Studies in healthy volunteers and pre-clinical studies shows that SMNP is a strong adjuvant that improves immunogenicity of different protein vaccines. The data of PWH is also reassuring that despite immune dysregulation activation of dendritic cells occur. HIV-specific immune responses were not directly evaluated in this study. The ex vivo data presented show that SMNP-mediated immune activation and cytokine-mediated latency reversal are not associated with cytotoxicity. However it’s impact on HIV-specific immune responses was not evaluated. It would strengthen the paper to include such evaluations, if possible.

Response: We agree with the reviewer, however due to technical limitations and limited sample availability this analysis has not been performed. Vaccine adjuvants are used to trigger innate response to boost recognition and triggering of downstream adaptive response against an antigen. Here we show that the adjuvants are capable of triggering an innate response using PBMC from PWH. This immune activation may also boost the autologous HIV-specific T cell response, however this is hard to determine in vitro, as peptide pools stimulation is the gold standard for the analysis of HIV-specific T cell responses bypassing major components of the innate immune response. The synthetic peptides pools are already processed thus skipping the complex intracellular processing pathways and innate danger-sensing mechanisms (like TLR and inflammasome activation) required for native proteins in vaccines.

Since effects on the HIV reservoir would be of interest in the context of therapeutic vaccination, the following statement in the abstract “However, as reservoir reduction was observed ex vivo, further evaluation is warranted to ensure safe use of such adjuvants in PWH” in unclear. It has been shown in studies of vaccination or co-infection that while viral blips may occur, during ART viral suppression is not compromised. The authors should clarify the statement about safety in the context of HIV and the broader goals of remission strategies.

Response: We have removed this sentence from the abstract, as this is not really addressed in this manuscript. We have included this in the introduction (line 90-93) and discussion (line 358-365).

Reviewer #2: The manuscript addresses whether MPLA and SMNP adjuvants induce innate immune activation in ART-suppressed PWH and if this lowers the inducible HIV-1 reservoir ex vivo. The conclusion that SMNP is more immunostimulatory than MPLA is supported by the activation and cytokine data and the observation that SMNP reduces inducible reservoir ex vivo has potential. The authors appropriately acknowledge that these are ex vivo findings requiring further evaluation before clinical translation. I ask that they also consider the following comments.

Major Comments

1. The statistical analysis section is too vague and not the best approach for the experimental design. The authors state that two-way ANOVA, one-way ANOVA, Brown-Forsythe/Welch ANOVA, and post-hoc corrections were used, but they do not clearly specify which test was used for each figure, whether donor-matched repeated-measures analyses were used, what correction was applied for each. Since many comparisons involve repeated measures across doses or treatments within the same donors, paired repeated-measures models or mixed-effects models would be more appropriate than simple ANOVA. Exact p values should be reported where possible and figure legends should clearly state the statistical test, n, and correction method for each panel.

Response: We have adjusted the statistical analysis as suggested by the reviewer and used a mixed-effect model to compare the different conditions within groups. This information has been added to the M&M section (line 187-191) and the legends of the figure. The exact p-values have been added to the figures and the number of donors/PWH tested is given in the figure legends.

2. I recommend more conservative language around reservoir reduction. The data suggest reduced inducible virus after SMNP exposure, but the mechanism is inferred rather than directly shown. The authors should avoid implying selective elimination of infected cells unless supported by additional evidence. Alternative explanations, including altered inducibility, assay interference, cytokine-mediated effects on outgrowth/readout, or changes in target-cell activation should be more explicitly discussed.

Response: We have adjusted our wording with regards to reservoir reduction throughout the manuscript and added alternative explanations for the observed reduction to the discussion (line 339-342).

3. The manuscript language is clear but would benefit from copy editing. There are multiple grammatical errors, spacing/formatting issues, and imprecise phrasing, including “ex vivowithout,” “supressed,” “monoplo,” and several awkward sentences in the Introduction and Discussion. These do not prevent interpretation but should be corrected.

Response: We have copy-edited the manuscript and corrected mistakes throughout the manuscript. We have rephrased parts in the introduction, results and discussion (line 57-64, 67-69, 213-216, 228-231, 240-244, 258-259, 269-275, 277, 281-283, 296-300, 314-319).

Attachment Submitted filename: Response to Reviewers.docx

# Peer review: Decision Letter 1

**DOI:** 10.1371/journal.pone.0348959.r003

**Authors:** Alan Landay

5 Jul 2026

Adjuvants MPLA and SMNP induce antiviral immunity and indirectly revert HIV-1 latency

PONE-D-26-18832R1

Dear Dr. Kootstra

We’re pleased to inform you that your manuscript has been judged scientifically suitable for publication and will be formally accepted for publication once it meets all outstanding technical requirements.

Within one week, you’ll receive an e-mail detailing the required amendments. When these have been addressed, you’ll receive a formal acceptance letter and your manuscript will be scheduled for publication.

An invoice will be generated when your article is formally accepted. Please note, if your institution has a publishing partnership with PLOS and your article meets the relevant criteria, all or part of your publication costs will be covered. Please make sure your user information is up-to-date by logging into Editorial Manager at Editorial Manager® and clicking the ‘Update My Information' link at the top of the page. For questions related to billing, please contact billing support.

If your institution or institutions have a press office, please notify them about your upcoming paper to help maximize its impact. If they’ll be preparing press materials, please inform our press team as soon as possible -- no later than 48 hours after receiving the formal acceptance. Your manuscript will remain under strict press embargo until 2 pm Eastern Time on the date of publication. For more information, please contact onepress@plos.org.

Kind regards,

Alan Landay

Academic Editor

PLOS One

Additional Editor Comments (optional):

Reviewers' comments:

Reviewer's Responses to Questions

**Comments to the Author**

1. If the authors have adequately addressed your comments raised in a previous round of review and you feel that this manuscript is now acceptable for publication, you may indicate that here to bypass the “Comments to the Author” section, enter your conflict of interest statement in the “Confidential to Editor” section, and submit your "Accept" recommendation.

Reviewer #1: All comments have been addressed

Reviewer #2: All comments have been addressed

**********

2. Is the manuscript technically sound, and do the data support the conclusions?

The manuscript must describe a technically sound piece of scientific research with data that supports the conclusions. Experiments must have been conducted rigorously, with appropriate controls, replication, and sample sizes. The conclusions must be drawn appropriately based on the data presented.

Reviewer #1: Yes

Reviewer #2: Yes

**********

3. Has the statistical analysis been performed appropriately and rigorously?

Reviewer #1: I Don't Know

Reviewer #2: Yes

**********

4. Have the authors made all data underlying the findings in their manuscript fully available?

The PLOS Data policy requires authors to make all data underlying the findings described in their manuscript fully available without restriction, with rare exception (please refer to the Data Availability Statement in the manuscript PDF file). The data should be provided as part of the manuscript or its supporting information, or deposited to a public repository. For example, in addition to summary statistics, the data points behind means, medians and variance measures should be available. If there are restrictions on publicly sharing data—e.g. participant privacy or use of data from a third party—those must be specified.

Reviewer #1: Yes

Reviewer #2: Yes

**********

5. Is the manuscript presented in an intelligible fashion and written in standard English?

PLOS ONE does not copyedit accepted manuscripts, so the language in submitted articles must be clear, correct, and unambiguous. Any typographical or grammatical errors should be corrected at revision, so please note any specific errors here.

Reviewer #1: Yes

Reviewer #2: Yes

**********

6. Review Comments to the Author

Please use the space provided to explain your answers to the questions above. You may also include additional comments for the author, including concerns about dual publication, research ethics, or publication ethics. (Please upload your review as an attachment if it exceeds 20,000 characters)

Reviewer #1: The authors have addressed my comments appropriately. I would recommend softening the statement in the abstract about a substantial reduction in the reservoir size. While the differences are statistically significant, these are ex vivo assays and it is far from clear that this level of reduction would have meaningful consequences in vivo. It is an interesting finding and of interest to the HIV remission field, however the statement could be modified to something like "reduced the size of the inducible reservoir ex vivo".

Reviewer #2: The authors have adequately addressed the concerns raised during the initial review. I recommend acceptance pending only minor editorial revisions.

**********

7. PLOS authors have the option to publish the peer review history of their article (what does this mean?). If published, this will include your full peer review and any attached files.

If you choose “no”, your identity will remain anonymous but your review may still be made public.

**Do you want your identity to be public for this peer review?**  For information about this choice, including consent withdrawal, please see our Privacy Policy.

Reviewer #1: **Yes:** Marina Caskey

Reviewer #2: **Yes:** Joshua Cyktor

**********

# Editor report: Acceptance letter

**DOI:** 10.1371/journal.pone.0348959.r004

**Authors:** Alan Landay

PONE-D-26-18832R1

PLOS One

Dear Dr. Kootstra,

I'm pleased to inform you that your manuscript has been deemed suitable for publication in PLOS One. Congratulations! Your manuscript is now being handed over to our production team.

At this stage, our production department will prepare your paper for publication. This includes ensuring the following:

* All references, tables, and figures are properly cited

* All relevant supporting information is included in the manuscript submission,

* There are no issues that prevent the paper from being properly typeset

You will receive further instructions from the production team, including instructions on how to review your proof when it is ready. Please keep in mind that we are working through a large volume of accepted articles, so please give us a few days to review your paper and let you know the next and final steps.

Lastly, if your institution or institutions have a press office, please let them know about your upcoming paper now to help maximize its impact. If they'll be preparing press materials, please inform our press team within the next 48 hours. Your manuscript will remain under strict press embargo until 2 pm Eastern Time on the date of publication. For more information, please contact onepress@plos.org.

You will receive an invoice from PLOS for your publication fee after your manuscript has reached the completed accept phase. If you receive an email requesting payment before acceptance or for any other service, this may be a phishing scheme. Learn how to identify phishing emails and protect your accounts at https://explore.plos.org/phishing.

If we can help with anything else, please email us at customercare@plos.org.

Thank you for submitting your work to PLOS ONE and supporting open access.

Kind regards,

PLOS ONE Editorial Office Staff

on behalf of

Prof. Alan Landay

Academic Editor

PLOS One
